# Understanding the Usage Patterns of Bicycle-Sharing Systems to Predict Users' Demand: A Case Study in Wenzhou, China

**DOI:** 10.1155/2018/9892134

**Published:** 2018-09-05

**Authors:** Xiaomei Xu, Zhirui Ye, Jin Li, Mingtao Xu

**Affiliations:** ^1^Jiangsu Key Laboratory of Urban ITS, Southeast University, Nanjing 210096, China; ^2^Jiangsu Province Collaborative Innovation Center of Modern Urban Traffic Technologies, Southeast University, Nanjing 210096, China; ^3^School of Transportation, Southeast University, Nanjing 210096, China; ^4^CCDI (Suzhou) Exploration & Design Consultant Co., Ltd., Suzhou 215123, China; ^5^Department of Transportation Engineering, School of Civil Engineering, Zhengzhou University, 100 Science Avenue, Zhengzhou, Henan 450001, China

## Abstract

Bicycle-sharing systems (BSSs) have become a prominent feature of the transportation network in many cities. Along with the boom of BSSs, cities face the challenge of bicycle unavailability and dock shortages. It is essential to conduct rebalancing operations, the success of which largely depend on users' demand prediction. The objective of this study is to develop users' demand prediction models based on the rental data, which will serve rebalancing operations. First, methods to collect and process the relevant data are presented. Bicycle usage patterns are then examined from both trip-based aspect and station-based aspect to provide some guidance for users' demand prediction. After that, the methodology combining cluster analysis, a back-propagation neural network (BPNN), and comparative analysis is proposed to predict users' demand. Cluster analysis is used to identify different service types of stations, the BPNN method is utilized to establish the demand prediction models for different service types of stations, and comparative analysis is employed to determine if the accuracy of the prediction models is improved by making a distinction among stations and working/nonworking days. Finally, a case study is conducted to evaluate the performance of the proposed methodology. Results indicate that making a distinction among stations and working/nonworking days when predicting users' demand can improve the accuracy of prediction models.

## 1. Introduction

Bicycle-sharing systems (BSSs) are usually used in two different ways: as an isolated service or as an intermodal service providing the missing link between existing points of other transport and desired destinations. Such systems are recognized to have environmental, traffic, and health benefits because they are emission-free, can augment public transport, and provide an incentive for athletic activity. In addition, they offer convenience for people who can use the service without the costs and responsibilities associated with owning a bicycle. DeMaio et al. indicated that there were three generations of BSSs, with the earliest dating back to the 1960s in Netherlands [1]. The “Witte Fietsen” (White Bikes) program was introduced on July 28, 1965, in Amsterdam, marking the initiation of the first-generation system. These white bikes were shared informally and not tracked, leading to numerous bike-thieves. In 1991, a coin-deposit system was introduced in Denmark, marking the initiation of the second-generation system. The third-generation system, which uses information technology to track bicycles and users, first emerged in the late 1990s in Europe and then extended to many cities all over the world. By the end of 2016, approximately 1,175 cities or districts in 63 countries have implemented BSSs, including 430 cities in China [2].

With the boom of BSSs, many bicycle-share related issues appear. A universal issue is the imbalance in spatial distribution of bicycles at stations over time due to disproportional usage patterns. This presents a challenge for people who may not be able to find a bicycle or an empty dock at certain stations, which will significantly reduce the service level of the system and the number of potential users. To satisfy users' needs, it is crucial to conduct rebalancing operations by removing bicycles from stations that are full and refilling the docks of empty stations. Nevertheless, redistributing bicycles directly according to the current state of the rental station may not be appropriate. The reason is that the pickup and return demands highly fluctuate throughout the day, which will make the redistributing strategy lag behind. As a result, demand prediction, specifying how many bicycles will be picked up or returned by users at a station during a predefined time interval in the future, is the key to rebalancing operations. As pickups and returns that happen simultaneously at the same station cancel each other out, the net values of pickups and returns during a predefined time interval in the future at each station are to be predicted in this paper. Based on the current state of the rental station and the predicted demand in the next interval, loading or unloading demand of each station at a specific moment can be determined for rebalancing operations.

Due to technical limitations, it is difficult to record every rental for the first- and second-generation systems. Although the third-generation systems can track the detailed rental data of bicycles, these data of most BSSs are not available to the public. Due to the lack of detailed rental data, many previous studies conducted research studies by using questionnaires [3, 4]. Bernatchez et al. recruited a sample of 7011 adults to conduct relative research studies on the BSS in Montreal [3]. Shaheen et al. issued two separate questionnaires to bike-sharing members and nonmembers to identify key differences and similarities between the two groups [4]. Some studies collected the real-time number of available bicycles at each station from the service website that intends to provide rental information for users to conduct research [5, 6]. Kaltenbrunner et al. recorded the number of available bicycles from the “Bicing” service website to conduct usage pattern analysis and demand prediction of the BSS in Barcelona [5]. Faghih-Imani et al. used the minute-by-minute number of bicycles available at each station saved from the bicycle-sharing service website in Montreal to calculate the arrival and departure rates [6]. As operators frequently perform rebalancing operations and the occurrence of these is not indicated within the data on number of bicycles available, it is impossible to directly distinguish whether the addition/removal of bicycles is due to users or operators. As more rental data goes public, there are more studies in the literature that apply the rental data to gain some insights into the BSSs. Two types of data are usually used in the literature: trip-based data and station-based data. Trip-based data (data capturing flows) are the rental data with every record representing a trip that has a pickup station/timestamp and a return station/timestamp. Station-based data (data capturing stocks) are trip-based data aggregated from the aspect of each station's pickups or returns, which can depict the stock fluctuation of each station. However, very few studies took reasonable measures to remove the unreal OD-C and OD-D trip records during the data processing phase (OD-C trip: trip's origin and destination stations are coincident; OD-D trip: trip's origin and destination stations are different.). Faghih-Imani et al. just removed all the OD-C trips without taking the trip duration into consideration [7]. Vogel and Zhou used the empirical duration threshold (60 seconds) to decide whether an OD-C or OD-D trip was real or not [8, 9].

In addition, methods to identify and visualize bicycle usage patterns have not been systematically or adequately examined in previous studies, particularly for large BSSs like Wenzhou. Most studies analyzed bicycle usage patterns from either trip-based aspect or station-based aspect unilaterally, and data on bad weather days (such as rain or snow conditions) were not removed [5, 6, 10]. As the time and location of rainy conditions will vary on different days, a weather factor like rainfall is a disturbing factor for the analysis of bicycle usage patterns. Some typical spatiotemporal usage patterns may not be found if the data on rainy days are used. Lines or curves are usually used to represent flows in the visualization of bicycle usage patterns, which works for systems with a small number of stations, but may not work for systems with a large number of stations. For example, Austwick et al. used opaque lines to represent flows of London BSS [11]. When the number of stations increases, these lines or curves may overlap and the underneath patterns will be eclipsed. Some practical methods, like sampling or showing a small subset of flow at a time [12], have been applied to resolve this difficulty. However, these approaches may either lose an overview of the patterns or miss some information conveyed by the whole dataset.

Existing prediction methods in the literature generally can be classified into two categories: factor analysis methods [6, 13–15] and time series methods [16–19]. Factor analysis methods are used to find factors (e.g., meteorological factors, calendar events, bicycle infrastructure, and land use) that have effects on bicycle usage and establish a functional relationship between them, as shown in the following equation:

(1) $$\begin{array}{c} \text{bicycle usage}=f\left(\text{meteorological factors, calendar events}\ldots \right). \end{array}$$

Since there are so many factors that influence bicycle usage, it is difficult to choose properly. Moreover, the data of some principal factors are difficult to collect or quantify, and the cost of data collection may be very high. Therefore, factor analysis methods have some limitations. Time series methods, intending to find the inherent law of historical data to establish prediction models, can be divided into linear methods, like ARIMA [16] and SARIMA [17], and nonlinear methods, like TAR [18] and BM [19]. These models usually have their own model assumptions. If any of the assumptions are inappropriate, the accuracy and reliability of results will be affected. Applying these methods into a complex and uncertain real-life case like BSS has numerous limitations. By comparison, using an artificial neural network (ANN) does not require any assumption to be made about the underlying function or model to be used or in-depth knowledge about inherent properties of the data [20]. This method has the advantages of self-learning, self-organization, self-adaptability, robustness, and generalization, which can solve complicated nonlinear problems with uncertainty [21]. Besides, many previous studies have proven that meteorological factors and calendar events have great influence on bicycle usage [22–24], and different regions with different built environments show different bicycle usage patterns [6, 8, 25]. However, existing studies seldom took all these factors into consideration when predicting users' demand.

In contrast to previous studies, the contributions of this study are as follows: (1) it proposes reasonable methods to remove the unreal OD-C and OD-D trip data, (2) it analyzes bicycle usage patterns from both trip-based aspect and station-based aspect, removing data on rainy days (which is a disturbing factor), (3) it introduces the concept of traffic zones from transportation planning to better visualize the bicycle flows, and (4) it makes a distinction among stations and working/nonworking days when constructing demand prediction models to improve prediction accuracy.

The remaining sections of this study are organized as follows.  Section 2 presents basic information about Wenzhou's BSS and introduces methods to collect and process the rental data.  Section 3 analyzes and visualizes the usage patterns of the BSS in Wenzhou. After that, the methodology to predict users' demand at each station on the basis of usage patterns analysis is presented in Section 4. Then, a case study is conducted to evaluate the performance of the proposed methodology in Section 5. Finally, the conclusions are summarized in the last section.

## 2. Data Collection and Processing

This section first presents basic information about Wenzhou's BSS. Then, methods to collect and process the data are discussed at length.

### 2.1. Overview of Wenzhou's BSS

**Figure 1:**
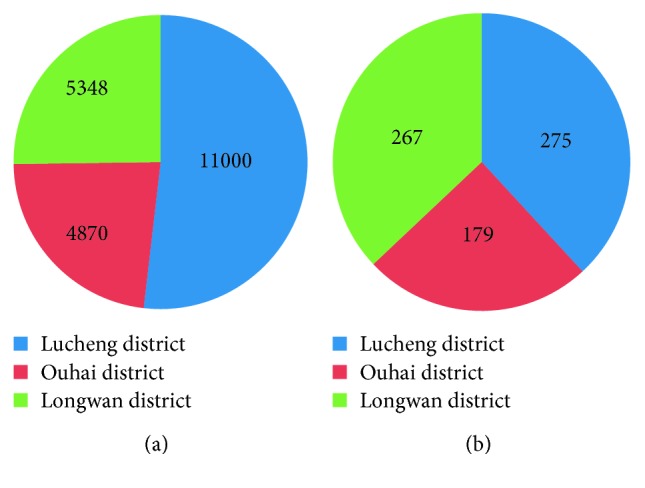
The number of bicycles and rental stations in different districts. (a) Bicycles and (b) rental stations.

**Figure 2:**
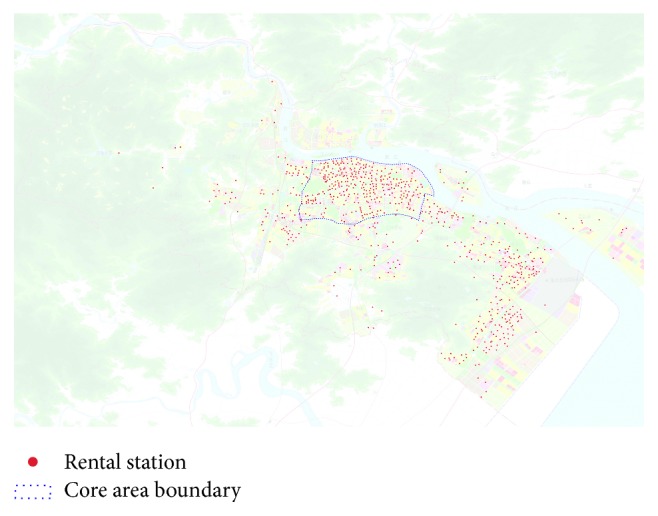
Spatial distribution of rental stations and the boundary of core area.

The BSS was first introduced to the Lucheng district of Wenzhou in 2012. By the end of 2016, there were 21,218 bicycles and 721 rental stations in the system. The distribution of bicycles and stations is depicted in Figures 1 and 2.  Figure 2 also outlines the core area, which is the heaviest traffic area in Wenzhou.

### 2.2. Data Collection

Three datasets were collected in this study, including the bicycle trip dataset, meteorological dataset, and calendar dataset. These datasets are integrated so that, for any single bicycle trip, it is possible to ascertain the weather condition (e.g., rainy or nonrainy) and whether it coincides with any salient calendar event (e.g., working or nonworking day).

**Figure 3:**
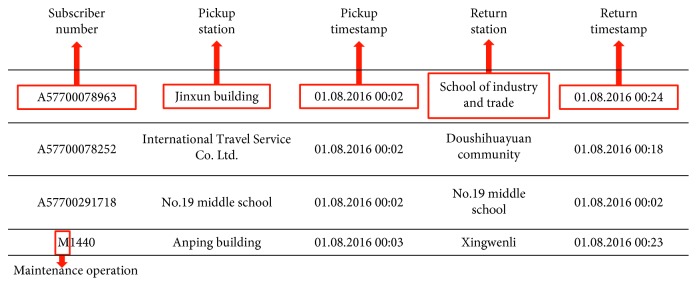
Structure of bicycle trip data.

Bicycle trip data from January to June and August to December in 2016 (July data were not recorded due to technical failures of the system) and the corresponding meteorological data are available to us. There are 18, 417, 507 bicycle trip records and 2,680 meteorological records. Meteorological data are collected at 2:00, 5:00, 8:00, 11:00, 14:00, 17:00, 20:00, and 23:00 every day. The raw bicycle trip dataset consists of ride information in the form of subscriber number, pickup station, and timestamp, as well as return station and timestamp, as shown in Figure 3. For each rental (trip), the system records the following:

1. The bicycle's pickup and return station.
2. Time of picking up and returning a bicycle (the minimum time unit is one minute.).
3. Subscriber number: the ID number of a card used to borrow a bicycle. A specific tag “M” tells whether the record is a user's rental or a maintenance operation.

### 2.3. Data Processing

Before a further analysis, datasets need to be cleaned and processed, according to the following procedure.

#### 2.3.1. Bicycle Trip Dataset

**Figure 4:**
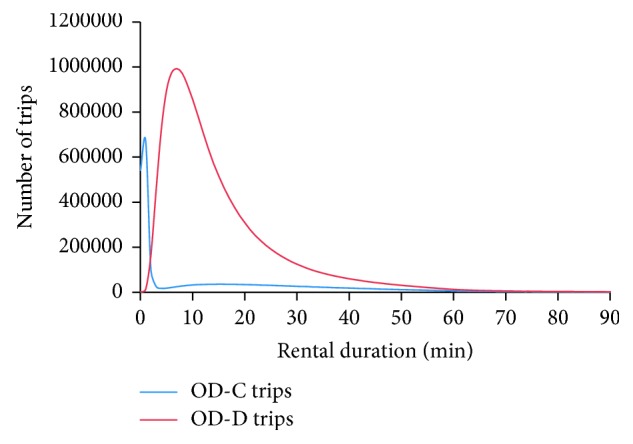
Frequency distributions of rental duration for OD-C and OD-D trips.

  Step 1: get rid of rental data due to maintenance operations.
  Rentals in the dataset are not all user based trips. It is important to note that BSS operators frequently perform maintenance operations, namely, removing bicycles from stations that are full and refilling the docks of empty stations. Rental records whose subscriber number begin with “M” indicate they are maintenance operations and should be removed.
  Step 2: remove incomplete records.
  Due to technical failures of the system, some parts of a few rental records are missing. These incomplete rental records should be removed.
  Step 3: calculate the duration of each rental.
  For each rental, subtracting the pickup timestamp from the return timestamp is the duration. The minimum unit of trip duration is one minute. Rental records with negative trip duration due to technical failures are removed.
  Step 4: remove unreal rentals.
  First, we divide the rentals into two types, OD-C trips and OD-D trips. OD-C trips with long duration are real rentals. However, OD-C trips with very short duration are not real rentals and these rental records should be removed. In this case, it is likely that the bicycle was returned to the same station immediately due to a mechanical fault, so the rider did not actually make the trip. The key is to find the trip duration threshold to distinguish between real and unreal OD-C trips. First, extract all the OD-C trips in the dataset and plot the frequency distribution of rental duration, as shown by the blue curve in Figure 4. For OD-C trips, the blue curve presents an extremely high frequency for trip duration *t* = 0 min, 1 min, and 2 min, whereas there is a large drop at *t* = 3 min, and the distribution is rather uniform for longer trips (*t* ≥ 3 min). It can be hypothesized that the two ranges of trips are typically different types. Therefore, this study regards OD-C trips shorter than 3 minutes (*t* = 0, 1, and 2 min) as unreal trips, and then these data should be removed. Furthermore, according to the definition in traffic engineering, a bicycle ride should be no shorter than 400 meters. That is, trips shorter than 96 seconds (bicycle speed *v* = 15 km/h) should be removed. Given these considerations above, OD-C trips that are shorter than 3 minutes (*t* = 0, 1, and 2 min) should be removed.
  With respect to the OD-D trips in the dataset, plot the frequency distribution of rental duration as shown by the red curve in Figure 4. Although a trip's origin and destination stations are different, a very short duration is possible. That is, a rider checks out a bicycle, then returns it to another extremely nearby station almost immediately due to a mechanical fault, so it does not count as a real trip. There are several pairs of stations of this kind, which are very extremely close together but have different names. For example, station “quzhengfudong” and station “quzhengfuxi” are two stations located on either side of the entrance of the district government office buildings, and the two stations are extremely close but with different names. For OD-D trips, the red curve presents an extremely low frequency for trip duration *t* = 0 min and 1 min, whereas there is a big leap at *t* = 2 min. Hence this study regards OD-D trips shorter than 2 minutes (*t* = 0 and 1 min) as unreal trips, so these data should be removed. Moreover, trips shorter than 96 seconds should be removed. Above all, OD-D trips that are shorter than 2 minutes (*t* = 0 and 1 min) should be removed.
  After removing the unreal OD-C and OD-D trips (almost 10% in the case of Wenzhou), we can finally obtain the real OD-C and OD-D trips in the dataset. These data are the final trip-based data used to conduct the following research in this study.
  Step 5: get station-based data.
  The processed data are trip-based valid data which are aggregated from the perspective of each pickup-return operation. If these data are aggregated from the aspect of each station's pickups or returns, we get the station-based data which can depict the stock fluctuation of each station.

In fact, some trips that could have happened have not happened, that is, rental stations were empty or full, which prevented users from borrowing or returning bicycles at the station. This represents that underlying trip demand has not been recorded by the BSS. In this paper, we ignore the underlying trip demand and consider the processed data as the true demand.

#### 2.3.2. Meteorological Dataset

**Figure 5:**
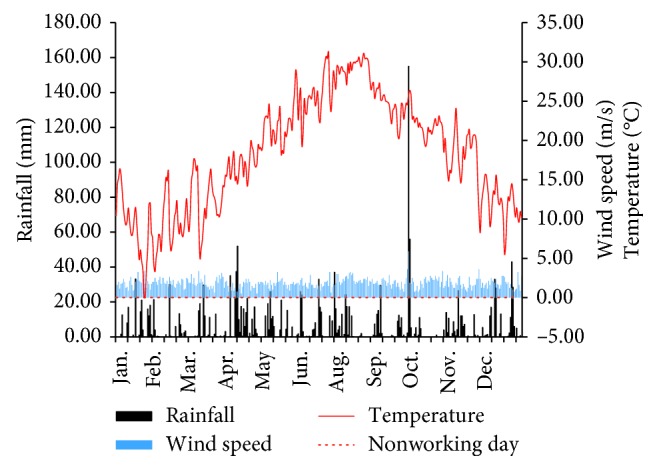
Daily value of meteorological indices and calendar events.

Main meteorological and calendar indices include wind speed, temperature, rainfall, and working/nonworking day. Meteorological data are collected at 2:00, 5:00, 8:00, 11:00, 14:00, 17:00, 20:00, and 23:00 every day. The arithmetic mean is computed to represent daily wind speed and temperature. The sum is calculated to represent daily rainfall. The daily value of wind speed, temperature, rainfall, and nonworking day is depicted in Figure 5.

## 3. Analysis of Bicycle Usage Patterns

There are two types of data, trip-based data and station-based data. Trip-based data can capture users' underlying mobility across the BSS network and station-based data can track variations in the number of bicycles docked at stations. Both types of data can help to shed light on the spatiotemporal usage patterns of the BSS.

### 3.1. Analysis on Trip-Based Data

Meteorological data, calendar events, bicycle infrastructure, land use, and built environment can influence bicycle usage patterns [26, 27]. The latter three are constant in the short run and thus they are not the emphasis of this research. Previous studies have revealed the significant roles weather and calendar events have played in cycling; the main weather/calendar indices include temperature, wind speed, rainfall, and working/nonworking day [24, 28]. To test whether or not these four indices have an influence on daily total trips in Wenzhou, correlation analysis was conducted. The results are presented in Table 1.

The results indicate that there is a strong negative correlation between daily bicycle usage and rainfall, as well as working/nonworking day. These results make sense because most people do not use bicycles on rainy days and want to rest at home when they do not have to work. Compared with rainfall and working/nonworking day, temperature appears to exert much less but positive influence on daily bicycle usage, and there is a very weak correlation between daily bicycle usage and wind speed. These results possibly highlight the relatively cycling-friendly climate, as there are not extremely cold or hot days in Wenzhou. This demonstrates that rainfall and working/nonworking day are the main factors that influence daily bicycle usage, and they should be considered when predicting users' demand.

#### 3.1.1. Temporal Patterns

**Figure 6:**
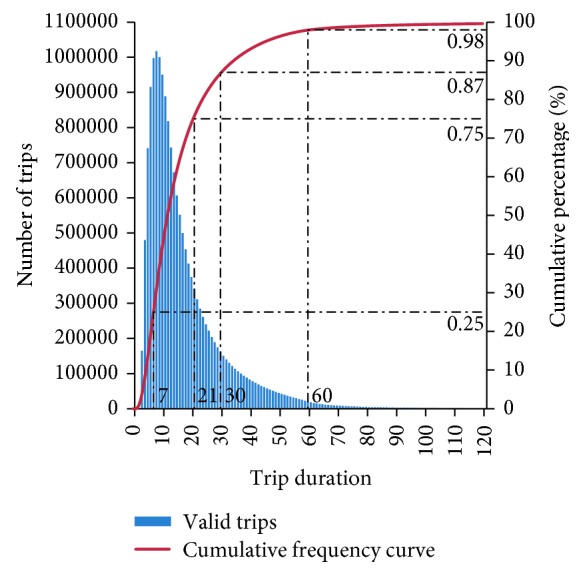
Rental duration distribution of valid trips.

The BSS system is designed for a quick turnaround of bicycles and the pricing structure discourages long-term rentals. Valid trip data are aggregated based on trip duration, as depicted in Figure 6. Most trips (87%) are less than 30 min. The third quartile is 21 min, and the interquartile range is 14 min, which verify that cycling is preferred for traveling a short time. Moreover, 98% trips are less than 60 min, which can be plausibly explained by the maximum minutes of free usage in Wenzhou.

#### 3.1.2. Spatial Patterns

**Figure 7:**
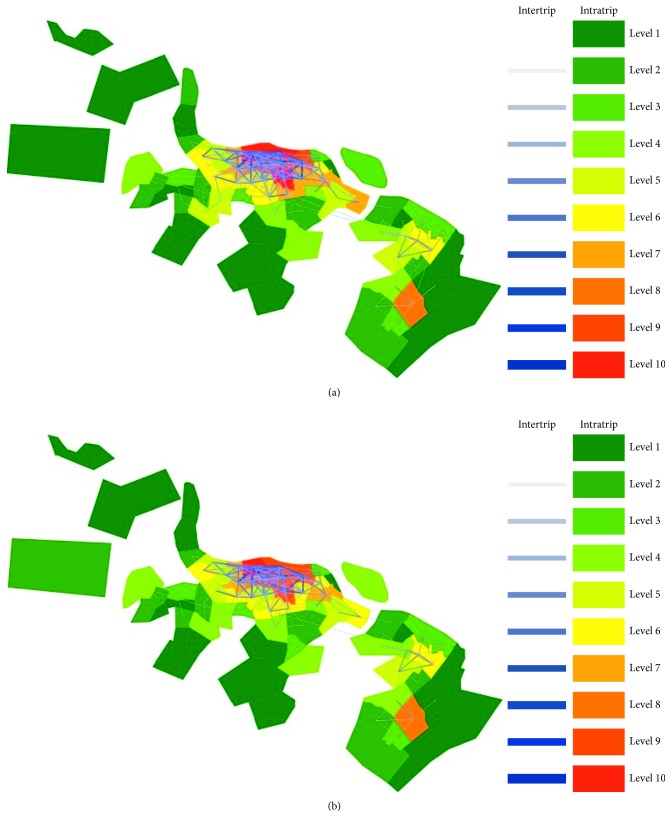
Daily bicycle trips between and within traffic zones. (a) Working days and (b) nonworking days.

Since there is a strong negative correlation between daily bicycle usage and rainfall as well as working/nonworking day, days of the whole year are divided into four types according to weather and calendar events: (1) working and nonrainy days, (2) working and rainy days, (3) nonworking and nonrainy days, and (4) nonworking and rainy days. As the time and location of rainy conditions will vary on different days, rainfall is a disturbing factor for the analysis of bicycle usage patterns. Typical spatiotemporal usage patterns may not be found if data on rainy days are considered. Besides, to enhance the capacity to visually depict the complex spatial flows, some aggregation of the spatial unit (from individual station to a traffic zone) is required. Several adjacent stations are grouped in a traffic zone leading to 83 traffic zones in total. As a result, valid trip data on nonrainy days are aggregated at traffic zone scale per day to analyze the spatial usage patterns. In addition, travel behavior on working days is different from that of nonworking days and hence BSS may show different usage patterns.  Figure 7 depicts the spatial bicycle flows on working and nonworking days separately at the traffic zone scale. The number of trips between traffic zones is represented by lines with variable thickness, while the number of trips within traffic zones is represented by different traffic zone colors (ten levels as shown in Figure 7, the higher levels represent the larger number of trips). The number of trips between or within traffic zones on working days is the average of the 107 working and nonrainy days. Similarly, the number of trips between or within traffic zones on nonworking days is the average of the 49 nonworking and nonrainy days.

As shown in Figure 7, the number of either intertrips (trips that take place between different traffic zones) or intratrips (trips that take place within a traffic zone) is very high in the core area where large-scale residential quarters and business districts are located, whereas the opposite case occurs in the surrounding suburb areas. This demonstrates that flows in different regions show different intensity. Additionally, both intertrips and intratrips have geographical concentration trends across the city, that is, the closer an area is to the center of core area, the larger the number of trips is. There is a high degree of interaction between adjacent traffic zones, and this highlights that bicycle trips usually take place over very short distances. The traffic flow on working days is heavier than that of nonworking days. For the number and distribution of trips in the suburb area, there is no obvious difference between working days and nonworking days. However, the number and distribution of trips in the core area on working days is quite different from that of nonworking days. This clarifies that the flows on different kinds of days show different intensity and distribution. Given that the flows in different regions or on different kinds of days show different intensity or distribution, it is necessary to make a distinction among stations and working/nonworking days when predicting users' demand.

### 3.2. Analysis on Station-Based Data

Rebalancing operations are station-based and hence it is necessary to break down the system-wide usage patterns to station-related pickup and return activities.

#### 3.2.1. Temporal Patterns

**Figure 8:**
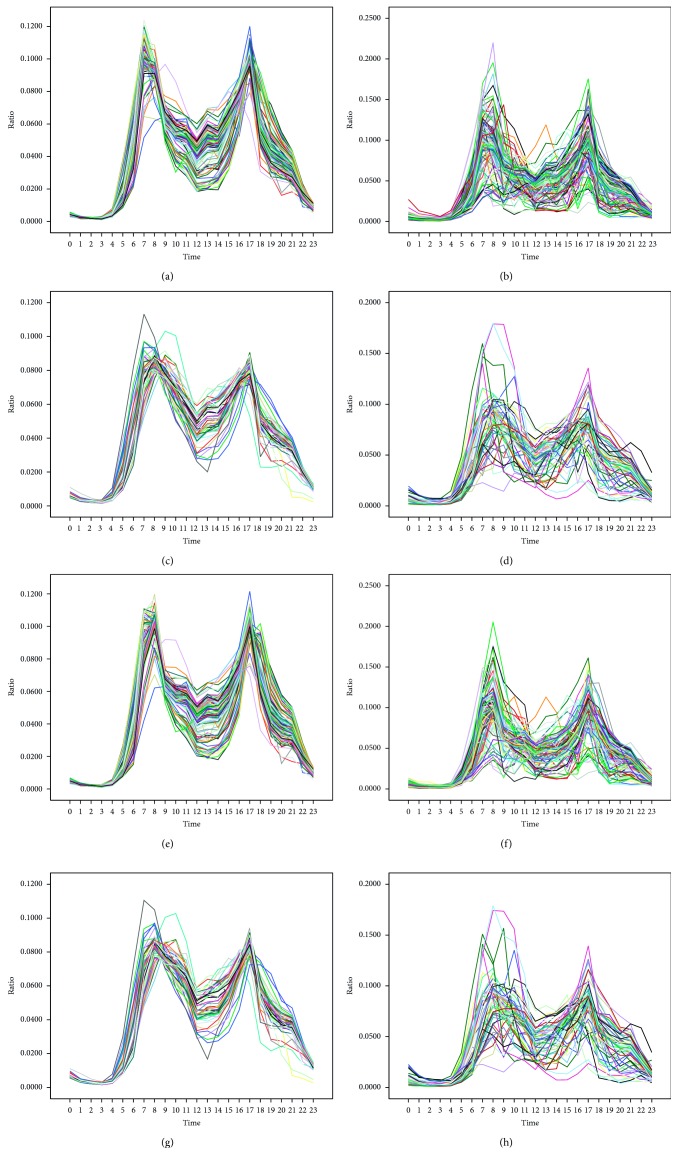
Pickup and return ratios for four different types of days. (a) Pickup on working and nonrainy days, (b) pickup on working and rainy days, (c) pickup on nonworking and nonrainy days, (d) pickup on nonworking and rainy days, (e) return on working and nonrainy days, (f) return on working and rainy days, (g) return on nonworking and nonrainy days, and (h) return on nonworking and rainy days.

The valid data are aggregated for 24 time windows per day, leading to 24 values representing all the stations' hourly pickups and 24 values representing all the stations' hourly returns every day. Normalization of the data is needed to compare the hourly fluctuation patterns of different days. The data are normalized by dividing the number of pickups or returns in a certain hour of a day by the total daily number.  Figure 8 shows the hourly fluctuation curve of pickup and return ratios every day for one year.

As shown in Figure 8, pickups and returns are very scarce between 00:00–5:00 AM, which will be a perfect time for night rebalancing operations to meet the optimal initial number of bicycles at every station.  Figure 8 reveals that hourly fluctuation patterns of different nonrainy days (working or nonworking) are highly consistent while that of different rainy days (working or nonworking) present the opposite case. This is because rain occurs at different time on different days, thus hourly fluctuation patterns of different rainy days (working or nonworking) are not consistent. This validates that rainfall is a disturbing factor for the analysis of BSS. Moreover, different kinds of nonrainy days (i.e., working and nonworking days) show different temporal pickup and return patterns: there are obvious morning and afternoon peaks on both working and nonrainy days (AM rush hour for pickups is 7:00–8:00 while PM rush hour for pickups is 17:00–18:00; AM rush hour for returns is 8:00–9:00 while PM rush hour for returns is 17:00–18:00) and nonworking and nonrainy days (AM rush hour for pickups is 8:00–9:00 while PM rush hour for pickups is17:00–18:00; AM rush hour for returns is 8:00–9:00 while PM rush hour for returns is 17:00–18:00). We find that the AM rush hour for pickups on working days is one hour earlier than that on nonworking days because most people do not have to get up as early on nonworking days. Additionally, the kurtosis of hourly fluctuation curves on nonworking days is not as distinct as working days, and the result is expected given that travel behavior and time are more scattered on nonworking days than working days. This analysis implies that a distinction should be made between working and nonworking days when predicting users' demand.

#### 3.2.2. Spatial Patterns

**Figure 9:**
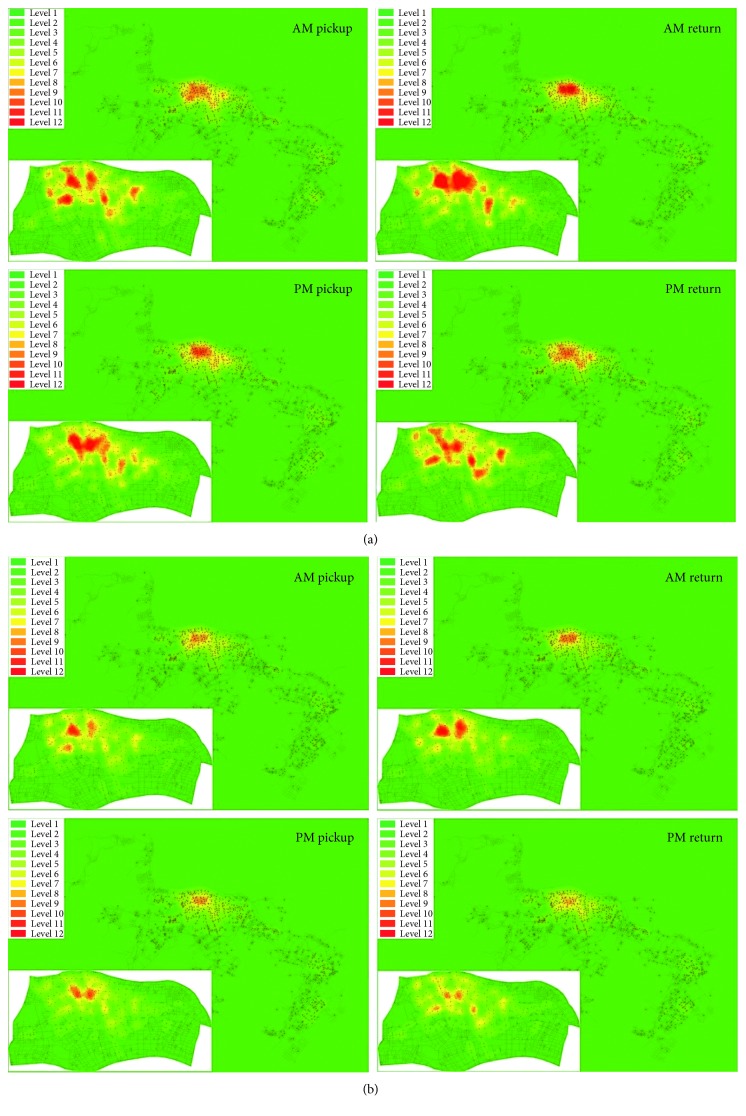
Pickups and returns in AM/PM rush hours. (a) Working days and (b) nonworking days.

Since the imbalance of bicycle distribution in BSSs is extremely severe during rush hours, Figure 9 presents each station's pickups and returns in AM/PM rush hours visually to better display the spatial variation of bicycle usage patterns in the system. The number of pickups and returns is represented by different colors (twelve levels as shown in Figure 9, higher levels represent a larger number of pickups and returns). The average hourly pickups and returns in AM/PM rush hours at every station across all 107 working and nonrainy days are computed to represent the working days, and in the same way, we can obtain the results for nonworking days.

Several interesting observations can be made from Figure 9. First, there are fewer pickups and returns during rush hours on nonworking days than on working days. Secondly, stations in different regions show different pickup and return intensity. On working days, pickups during AM rush hours are mainly concentrated in several dispersed residential quarters while returns during AM rush hours are mainly concentrated in the central business district, and the opposite case occurs in PM rush hours. This confirms the use of BSS for daily commute purposes. In the AM/PM rush hours of nonworking days, pickups and returns show obvious aggregation features only in two main residential quarters. Thirdly, pickups and returns during PM rush hours are slightly fewer than that during AM rush hours on working or nonworking days. This can be plausibly explained by people's pressing desire to get home as early as possible. These individuals might decide to go home by other faster transportation modes. The above analysis demonstrates that a distinction should be made among stations and working/nonworking days when predicting users' demand.

## 4. Methodology for Demand Prediction

Analyzing spatiotemporal usage patterns of a BSS provides essential information for the establishment of users' demand prediction models. According to the spatiotemporal analysis in previous sections, a distinction should be made among rental stations and working/nonworking days when predicting users' demand. Moreover, rainfall should be considered. Here, cluster analysis is applied to distinguish different station types. Given that one station may show different patterns on working days and nonworking days, cluster analysis should be conducted on working days and nonworking days, respectively. By considering calendar events (i.e., working and nonworking days) and station types (*m* types for working days and *n* types for nonworking days), different back-propagation neural networks are established to predict users' demand. Since rainfall is a disturbing factor, only the data on nonrainy days are utilized in cluster analysis and network training.

### 4.1. Cluster Analysis

When the hourly fluctuant patterns of pickups and returns are analyzed at individual station level, different service types of stations can be identified. Here, cluster analysis is used to group stations according to their hourly pickup and return ratio. Cluster analysis or clustering is the task of grouping a set of objects in such a way that objects in the same cluster are more similar to each other than to those in other clusters. Bicycle data on nonrainy days are applied for cluster analysis on working days and nonworking days, respectively. To determine whether or not there is a nonrandom structure in the dataset, the Hopkins statistic [29] is used to measure the cluster tendency. A statistic result close to 1 indicates the dataset is highly clustered. With respect to the random datasets, the statistic result is around 0.5. The corresponding result is close to 0 for uniformly distributed data.

As the dataset is very large, the K-means algorithm which has a good convergence rate is applied. The K-means algorithm aims to partition *n* observations into *k* clusters in which each observation belongs to the cluster with the nearest mean. The behavior of the K-means algorithm depends on the input parameters (i.e., the clustering number *k* and the initial clustering centers). Only specific values of the algorithm's input parameters can lead to optimal partition. The specific process of applying the K-means algorithm to conduct cluster analysis is provided as follows:

Step 1: initialize input parameters.
For the K-means algorithm, the clustering number *k* and the initial clustering centers are two parameters that need to be determined. Here *k* varies from two to ten. The maximum-minimum principle is used to determine the initial clustering centers for each *k* and the specific process is provided as follows: first, find two points (*x*_1_, *x*_2_) from the dataset with the largest Euclidean distance. The third point *x*_3_ is then explored by maximizing the lesser of the Euclidean distance (*x*_3_, *x*_1_) and the Euclidean distance (*x*_3_, *x*_2_). Repeat the above step until all the *k* points are found and (*x*_1_, *x*_2_,…, *x*_*k*_) are the initial clustering centers.
Step 2: for each *k* and the corresponding initial clustering centers (*x*_1_, *x*_2_,…, *x*_*k*_), apply the K-means algorithm to obtain different clustering results.
Step 3: compute the *S*_Dbw validation index of each partition in step 2.
A clustering validation index “*S*_Dbw” is deployed to determine the optimal clustering number *k* (varying from two to ten). Clustering validation is an essential step to determine the success of a clustering application. Clustering aims to group the objects with similar patterns within the same cluster. Therefore, clustering validation should adhere to two criteria, cohesion and separation. Cohesion represents the degree of similarity for the objects in a cluster and separation reflects the degree of distinction for the objects in different clusters. Many indices for clustering validation have been proposed in previous studies. Some of them only consider one criterion (e.g., root-mean-square standard deviation and R-squared [30]) whereas some consider both (e.g., Dunn's index [31], Davies–Bouldin index [32], and *S*_Dbw index [33]). Liu et al. [34] investigated the validation properties of 11 widely used clustering validation indices from five conventional aspects of clustering. The results indicated that *S*_Dbw was the only validation index that performed well in all five aspects while others had certain limitations in different application scenarios. Consequently, *S*_Dbw is adopted to conduct clustering validation in this study, as shown in the following equation:
  (2) $$\begin{array}{c} S\text{\_Dbw}\left(k\right)=\text{Scat}\left(k\right)+\text{Dens}\_\text{bw}\left(k\right),\\ \text{Scat}\left(k\right)=\frac{1}{k}\overset{k}{\underset{i=1}{\sum }}\frac{\left\|\sigma \left(v_{i}\right)\right\|}{\left\|\sigma \left(S\right)\right\|},\\ \text{Dens\_bw}\left(k\right)=\frac{1}{k\cdot \left(k-1\right)}\overset{k}{\underset{i=1}{\sum }}\left\{\overset{k}{\underset{j=1,i\neq j}{\sum }}\frac{\text{density}\left(u_{ij}\right)}{\max\left\{\text{density}\left(v_{i}\right),\text{density}\left(v_{j}\right)\right\}}\right\}, \end{array}$$
  where *v*_*i*_ is the center of cluster *i*; *S* is the dataset; *σ*(*v*_*i*_) and *σ*(*S*) are the variance of cluster *i* and the dataset, respectively; *u*_*ij*_ is the middle point of the line segment defined by the two clustering centers *v*_*i*_ and *v*_*j*_; and density(*v*_*i*_), density(*v*_*j*_), and density(*u*_*ij*_) represent the density of cluster *i*, cluster *j*, and the area between cluster *i* and *j*.
For more specific information about the *S*_Dbw, refer to Halkidi and Vazirgiannis [33]. As shown in Equation (2), both cohesion (Scat(*k*)) and separation (Dens_bw(*k*)) criteria are properly considered in the *S*_Dbw, and this will enable a reliable clustering validation. A small Scat(*k*) value is an indication of compact clusters. A small Dens_bw(*k*) value shows well-separated clusters. Therefore, a small *S*_Dbw value indicates a reasonable clustering.
Step 4: determine the optimal clustering number and the best partition according to the value of the *S*_Dbw validation index computed in step 3.

### 4.2. Back-Propagation Neural Networks

To predict users' demand, different back-propagation neural networks are respectively established for each station type on working days and nonworking days.

**Figure 10:**
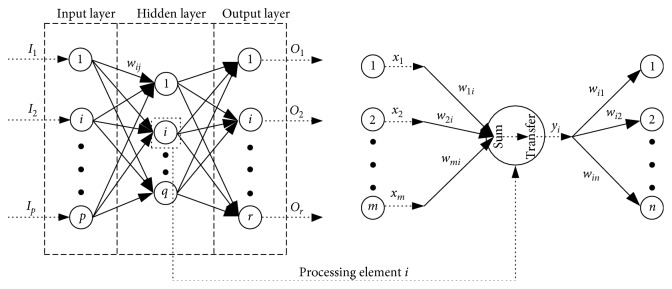
Geometry and operation of a typical back-propagation neural network.

An artificial neural network (ANN) is a parallel-computing system that consists of many neurons, emulating the ability of a biological neural network [35]. There are many types of ANN systems with different algorithms. One of the most popular is the back-propagation neural network (BPNN). A BPNN is a layered network consisting of an input layer, an output layer, and at least one hidden layer [36]. Since adjusting the number of neurons per hidden layer is more preferable than increasing the number of hidden layers, a three-layered network (as shown in Figure 10) with only one hidden layer is adopted in this study. Different layers, composed of several neurons, are interconnected by sets of connection weights. From Figure 10, we can note that every neuron in the hidden and output layer can be seen as a processing element which has two functions (i.e., sum and transfer). The input signal (*I*_1_, *I*_2_, …, *I*_*p*_) flows through the whole network, producing an ultimate output signal (*O*_1_, *O*_2_,…, *O*_*r*_), which is a function of the connection weights (*w*_*ij*_), the transfer function, and the specific network geometry (i.e., the number of hidden layers and the number of neurons per hidden layer).

The purpose of training a BPNN is to capture the relationship (connection weights) between the input and output signal by repeatedly presenting historical examples of input/output signals to the network. There are two general steps in the training process of a BPNN. *The first step* is a feed-forward iteration to calculate the values of ultimate output signal (*O*_1_, *O*_2_,…, *O*_*r*_) in the network. This calculation is based on input values of the input signal (*I*_1_, *I*_2_, …, *I*_*p*_). For each processing element *i*, the output values of different neurons in the previous layer (*x*_1_, *x*_2_, …, *x*_*m*_) are multiplied by the corresponding connection weights (*w*_1*i*_, *w*_2*i*_,…, *w*_*mi*_). The weighted values (*w*_1*i*_ · *x*_1_, *w*_2*i*_ · *x*_2_,…, *w*_*mi*_ · *x*_*m*_) are summed and then a threshold value (*θ*_*i*_) is added. The combined value (*I*_*i*_) is then passed through a transfer function to produce the output value (*y*_*i*_) of the processing element *i*. This output value (*y*_*i*_) acts as the input value for neurons in the next layer. This process is concretely illustrated in Equation (3) and Figure 10. For processing element *i*,

(3) $$\begin{array}{c} \text{sum}:I_{i}=\overset{m}{\underset{k=1}{\sum }}w_{ki}\cdot x_{k}+\theta_{i},\\ \text{transfer}:y_{i}=f\left(I_{i}\right), \end{array}$$

where  *f*(·)  is  the  transfer  function.

*The second step* is the back-propagation. It is an iterative gradient descent algorithm designed to minimize the error between the predicted output values (the calculated values of ultimate output signal (*O*_1_, *O*_2_,…, *O*_*r*_) in the first step, *y*_*i*_) and the training dataset values (desirable or historical output values, *d*_*i*_) by adjusting the connection weights. The most commonly used error is the mean squared error (MSE) expressed as *E*=(1/*r*)∑_*i*=1_^*r*^(*y*_*i*_ − *d*_*i*_)^2^, where *r* is the number of neurons in the output layer. The error *E* will propagate backwards to the input layer through the hidden layer. A gradient descent algorithm is utilized to adjust the connection weights repeatedly so as to minimize the output error *E*. The gradient descent algorithm adapts the weights according to the gradient error. Buscema indicated that the adjustment amount of connection weight between neuron *i* and *j*, Δ*w*_*ij*_, can be estimated by the following equation [37]:

(4) $$\begin{array}{c} \Delta w_{ij}=\eta \cdot \delta_{j}^{n}\cdot y_{i}^{n-1},\\ \delta_{j}^{n}=\left\{\begin{array}{cc} \left(d_{j}-y_{j}\right)\cdot y_{j}\cdot \left(1-y_{j}\right), & \left(j\text{ is in the output layer}\right),\\ \left[\overset{r}{\underset{k=1}{\sum }}\delta_{k}^{n+1}\cdot w_{jk}\right]\cdot y_{j}\cdot \left(1-y_{j}\right), & \left(j\text{ is in the hidden layer}\right), \end{array}\right. \end{array}$$

where *η* is the positive-learning constant; *y*_*i*_^*n*−1^ is the output value of neuron *i* in the layer before neuron *j* that is related to the connection weight *w*_*ij*_; *n*, *n* − 1, and *n*+1, respectively, represent the current layer where *j* is, the layer before *j*, and the layer after *j*; *d*_*j*_ is the desirable output value of neuron *j* in the output layer; *y*_*j*_ is the calculated output value of neuron *j*; and *r* is the number of neurons in the output layer.

Rumelhart et al. pointed out that the momentum term with a momentum gain *α* can accelerate the convergence of the error back-propagation learning algorithm [38]. Consequently, the value of the connection weight can be expressed as

(5) $$\begin{array}{c} w_{ij}^{m}=w_{ij}^{m-1}+\eta \cdot \delta_{j}^{n}\cdot y_{i}^{n-1}+\alpha \cdot \Delta w_{ij}^{m-1}, \end{array}$$

where *α* is a user-selected positive momentum constant and *m* and *m* − 1 separately signify the current and previous training step.

**Figure 11:**
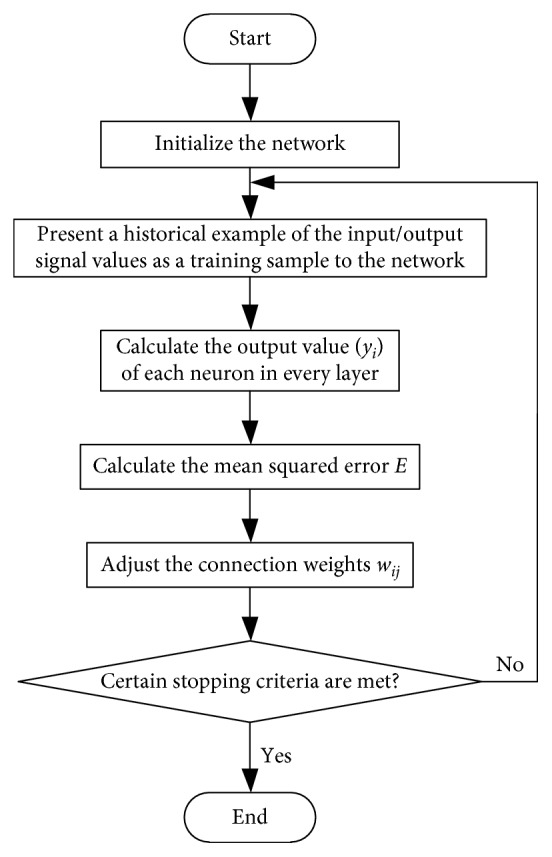
Training process of a back-propagation neural network.

The training process of a BPNN is summarized in Figure 11. Training is usually continued until certain stopping criteria are satisfied. For example, training may be stopped when the mean squared error *E* is sufficiently small or there is no further improvement of *E* when increasing the epoch (the number of steps in the training process).

If the rainfall of the time interval during which we predict is 0, the corresponding back-propagation neural network is then used to predict the demand. If the rainfall during the time interval which we predict is not 0, a reduction (*R*_*i*_) in proportion to the rainfall will be subtracted from the value of the back-propagation neural network calculated, as depicted in the following equation:

(6) $$\begin{array}{c} R_{i}=s\cdot p\cdot \frac{C_{i}}{\sum_{i=1}^{721}C_{i}}, \end{array}$$

where *R*_*i*_ is the reduction amount to be subtracted of station *i*; *s* is the elastic coefficient of rainfall; *p* is the rainfall in the time interval during which we predict the demand; and *C*_*i*_ is the number of parking docks of station *i*.

### 4.3. Comparative Analysis

Comparative analysis is employed to determine if the accuracy of the prediction models is improved by making a distinction among stations and working/nonworking days. The back-propagation neural networks are trained under different scenarios:

  Scenario 1: only make a distinction between working days and nonworking days
  Scenario 2: only make a distinction among stations, and
  Scenario 3: make a distinction among stations and working/nonworking days

We used the real-life case of Wenzhou to compare the prediction accuracy of different scenarios.

## 5. Case Study

In this section, the real-life case of Wenzhou in China is selected to evaluate the performance of the proposed methodology.

### 5.1. Cluster Analysis of Stations

Data of 107 working and nonrainy days were used for cluster analysis on working days and data of 49 nonworking and nonrainy days were utilized for cluster analysis on nonworking days. The data were aggregated for 24 time windows per day at individual station level, leading to 24 values representing the hourly pickups and 24 values representing the hourly returns for every individual station per day. For each station, we computed the average hourly pickups/returns across the 107 working and nonrainy days and across the 49 nonworking and nonrainy days, respectively, leading to 48 values (24 values for pickups and 24 values for returns) representing the usage pattern on working days and nonworking days, respectively. Normalization of the data is needed. For every individual station, the data were normalized by dividing the number of pickups or returns in a certain hour by the total daily number.

**Figure 12:**
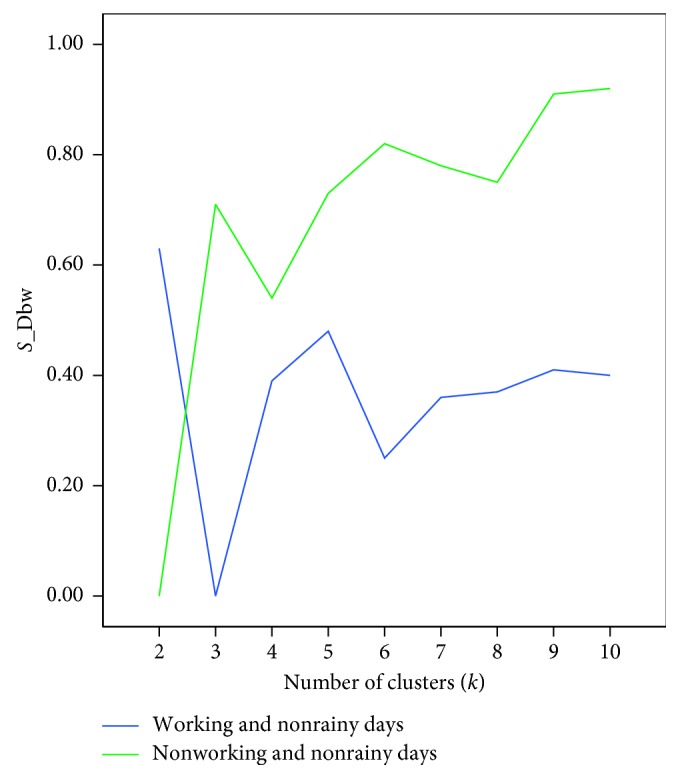
*S*_Dbw for working and nonworking days.

The Hopkins statistic was then applied to the normalized data. The statistic results for working days and nonworking days are, respectively, 0.81 and 0.73, which indicates that these two datasets are suitable for clustering. The K-means algorithm was applied to the datasets of working days and nonworking days, respectively, with the clustering number *k* ranging from 2 to 10. The behavior of *S*_Dbw is presented in Figure 12.

**Figure 13:**
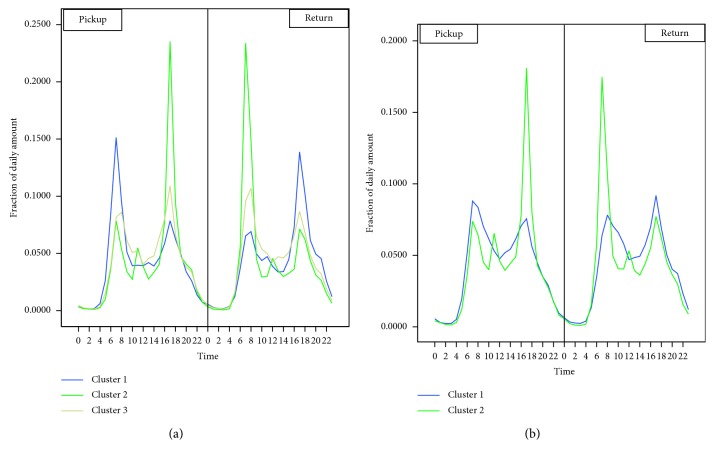
Final clustering centers for (a) working and (b) nonworking days.

As we can see from Figure 12, the reasonable clustering number for working days and nonworking days is *k*=3 and *k*=2, respectively. The final clustering centers are shown in Figure 13.

### 5.2. Demand Prediction of Stations (Network Training and Testing)

The net value of pickups and returns during a predefined time interval in the future (prediction interval *t*) at each station is predicted in this paper. This value termed as *d*(*t*), is the only variable of the output layer in the BPNN. The variables of the input layer in the BPNN include:

1. The net value of pickups and returns during time interval *t* − 1, *t* − 2, and *t* − 3, that is *d*(*t* − *i*), *i*=1, 2, 3. *t* − 1, *t* − 2, and *t* − 3 are the first, second, and third time intervals before the prediction interval *t*.
2. The net value of pickups and returns during time interval *tt*, that is, *d*(*tt*). *tt* is the same time interval as the prediction interval *t* but in the previous week.
3. The net value of pickups and returns during time interval *tt* ± 1, *tt* ± 2, and *tt* ± 3, that is, *d*(*tt* ± *i*), *i*=1, 2, 3. *tt* − 1, *tt* − 2, and *tt* − 3 are the first, second, and third time intervals before the time interval *tt*. *tt* ± 1, *tt* ± 2, and *tt* ± 3 are the first, second and third time intervals after the time interval *tt*.

Datasets of Wenzhou were chosen for network training and testing. Pickups and returns at each station were aggregated every 10 minutes, and the net value was then calculated, leading to 144 values representing each station's net value of pickups and returns every day. Each value (*d*(*t*)) was matched with 10 corresponding values (*d*(*t* − *i*), *d*(*tt*), *d*(*tt* ± *i*), *i*=1, 2, 3), serving as one sample for network training and testing. The network was composed of an input layer with ten input variables, a hidden layer with eight nodes, and an output layer with one output variable. The BPNN was used to train the datasets. The matched dataset was randomized, and one sample was selected for every four samples; these two parts were used to build a training dataset and a testing dataset. The software Neurosolutions, Version 7.1.1.1 was used to simulate neural network operation using an Intel(R) Core(TM) i3-2330 M CPU @ 2.20 GHz PC.

**Figure 14:**
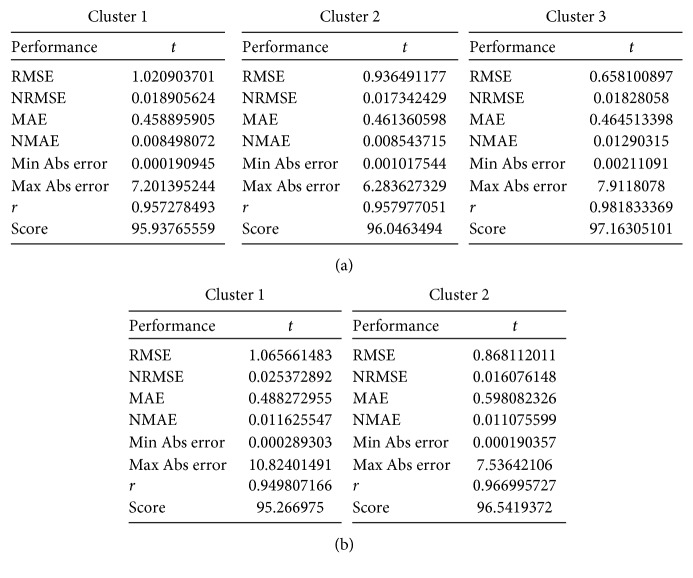
Performance of BPNNs. (a) Working days and (b) nonworking days.

**Figure 15:**
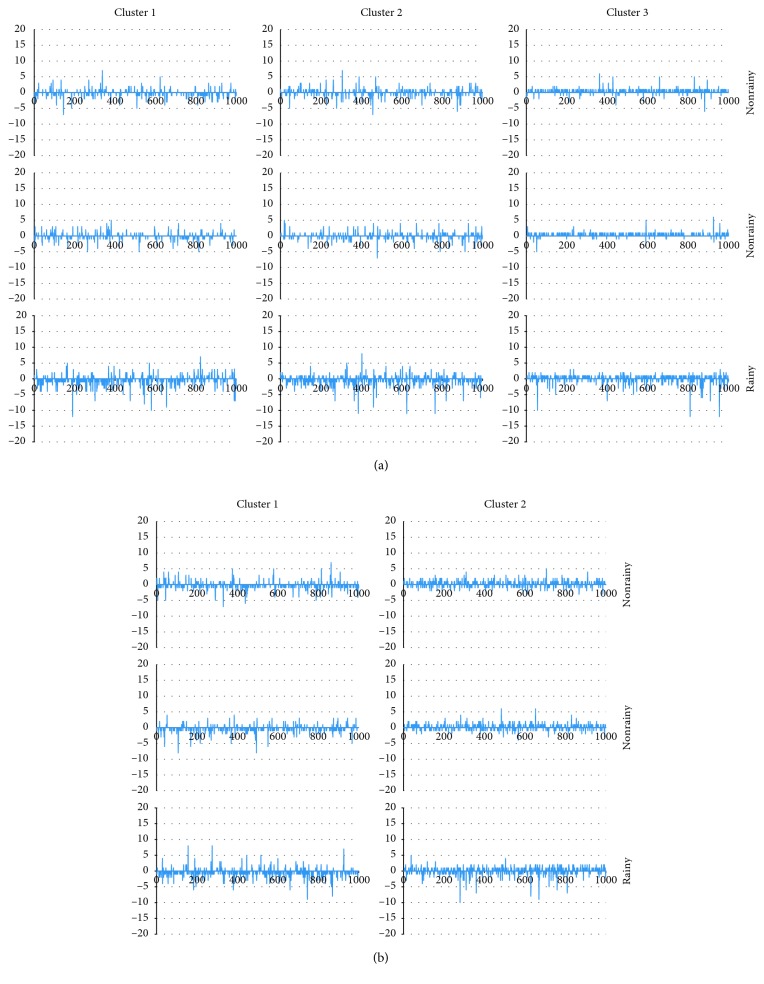
Prediction error of random trials. (a) Working days and (b) nonworking days.

Three BPNNs were established for working days while two were established for nonworking days, respectively. To calculate the elastic coefficient of rainfall *s*, “mfx” order was used after negative binomial regression analysis between daily total trips and the four factors (i.e., wind speed, temperature, rainfall, and working/nonworking day) in STATA, *s*=577. The performance of each network is presented in Table 2 and Figure 14. The coefficient *R*^2^ was used to evaluate the accuracy of network training. After network testing, the *R*^2^ value of different networks were all above 0.9. The largest mean absolute error (MAE) and root mean squared error (RMSE) are 0.60 and 1.07, respectively, denoting that the trained BPNNs can describe the experimental data with a good accuracy. New data samples were deployed to further test the prediction accuracy of the proposed methodology. For each cluster, three groups of 1,000 random samples (two groups are on nonrainy days and one group is on rainy days) were introduced into the trained network, and the results for calculation of prediction error (“Prediction error” is the error between the predicted output values and the historical output values, and “Abs error” is the absolute value of prediction error.) are presented in Figure 15 and Table 3. As we can see, the percentage of “Abs error = 0” exceeds 70% for every random trial, and the number goes up to around 90% for the percentage of “Abs error ≤ 1.” This implies that the proposed methodology can achieve very good performance in predicting users' demand. The percentages of “Abs error = 0” and “Abs error ≤ 1” on nonworking days are both a little lower than those on working days; the percentages of “Abs error = 0” and “Abs error ≤ 1” on rainy days are both a little lower than those on nonrainy days. This implies the prediction accuracy on nonworking days is a little lower than that on working days, and the prediction accuracy on rainy days is a little lower than that on nonrainy days. These results make sense as travel behavior is more uncertain on nonworking days or rainy days, which presents a challenge for demand prediction.

### 5.3. Comparative Analysis under Different Scenarios

**Figure 16:**
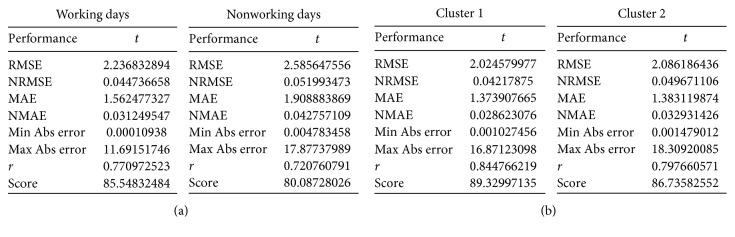
Performance of BPNNs under Scenario 1 and Scenario 2. (a) Scenario 1 and (b) Scenario 2.

**Figure 17:**
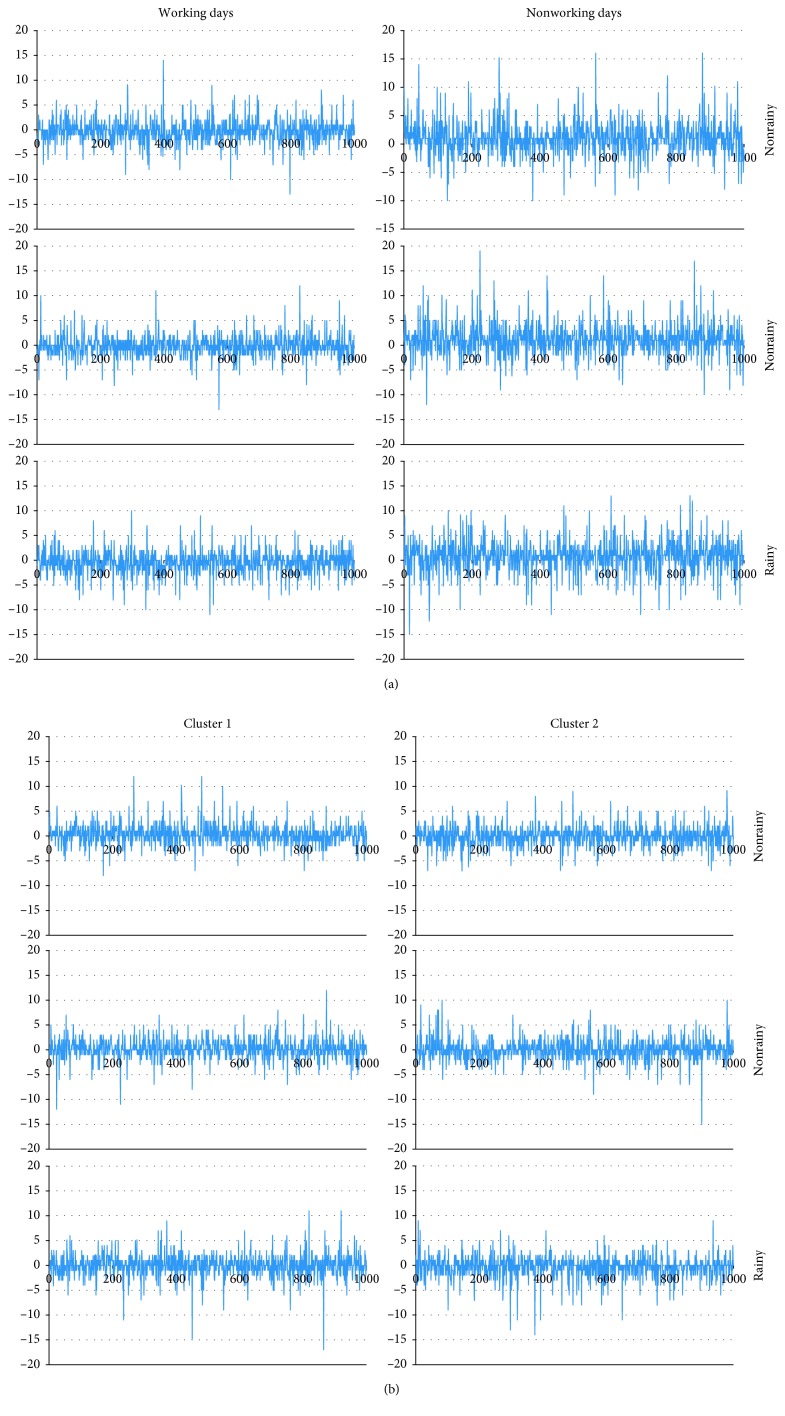
Prediction error of random trials under (a) Scenario 1 and (b) Scenario 2.

To determine if the accuracy of the prediction models is improved by making a distinction among stations and working/nonworking days, back-propagation neural networks were trained under Scenario 1 (which only makes a distinction between working days and nonworking days) and Scenario 2 (which only makes a distinction among stations) separately by using the real-life case of Wenzhou. The results are presented in Tables 4 and 5, and Figures 16 and 17. In Scenario 1, the *R*^2^ value of working days is 0.59 and the *R*^2^ value of nonworking days is 0.52; the percentage of “Abs error = 0” is around 25% for all random trials of working days and the number is around 60% for the percentage of “Abs error ≤ 1”; the percentage of “Abs error = 0” is less than 15% for all random trials of nonworking days and the number is less than 50% for the percentage of “Abs error ≤ 1.” In Scenario 2, the *R*^2^ value of cluster 1 is 0.71, and the *R*^2^ value of cluster 2 is 0.64; the percentage of “Abs error = 0” is around 30% for all random trials of cluster 1 and the number is around 65% for the percentage of “Abs error ≤ 1”; the percentage of “Abs error = 0” is less than 35% for all random trials of cluster 2 and the number is less than 65% for the percentage of “Abs error ≤ 1.”

Finally, we compared the results in Tables 4 and 5 and Figures 16 and 17 (Scenario 1 and Scenario 2) with those in Tables 2 and 3 and Figures 14 and 15 (Scenario 3: make a distinction among stations and working/nonworking days.). The *R*^2^ value of Scenario 1 and Scenario 2 is far less than that of Scenario 3, and the Abs error of Scenario 1 and Scenario 2 is much larger than that of Scenario 3. This implies that making a distinction among stations and working/nonworking days when predicting users' demand can improve the accuracy of prediction models.

## 6. Conclusions and Future Work

Based on the detailed rental data of the BSS in Wenzhou, China, this study proposed users' demand prediction models, which are crucial to the success of rebalancing operations. Bicycle usage patterns were first analyzed to shed light on the characteristics of the BSS. The results revealed that bicycle usage patterns on working days were largely different from those on nonworking days, and stations in different regions with different built environments showed different usage patterns. A distinction should be made among stations and working/nonworking days when predicting users' demand. Then, cluster analysis was used to identify different service types of stations on working and nonworking days, that is, three types for working days and two types for nonworking days. The BPNN method was then used to establish different demand prediction models according to different types of stations and days. After that, comparative analysis was employed to determine if the performance of prediction models improves by making a distinction among stations and working/nonworking days. The analysis results revealed that the *R*^2^ value of Scenario 1 and Scenario 2 is far less than that of Scenario 3, and the Abs error of Scenario 1 and Scenario 2 is much larger than that of Scenario 3, which implied that making a distinction among stations and working/nonworking days when predicting users' demand can improve the accuracy of prediction models.

This research did not address underlying users' demand, which is the number of trips of users who are prevented from borrowing a bicycle at empty stations and the number of trips of users who are prevented from returning a bicycle to a full station. Further study on the underlying demand needs to be conducted. Users' demand prediction and the development of rebalancing models and algorithms are two crucial parts in the rebalancing of a bicycle-sharing system. Users' demand prediction which accommodates a heavy workload is the research emphasis in this paper. Future study on the rebalancing model and algorithm needs to be conducted.

## Figures and Tables

**Table 1:** Bivariate correlate and partial correlate results.

			Temperature	Wind speed	Rainfall	Working/nonworking day
Bivariate correlate	Daily total trips	Correlation	0.332^*∗∗*^	0.126^*∗*^	−0.607^*∗∗*^	−0.504^*∗∗*^
		Significance (2-tailed)	0.000	0.021	0.000	0.000
		df	335	335	335	335

Partial correlate	Daily total trips	Correlation	0.354	0.161	−0.625	−0.518
		Significance (2-tailed)	0.000	0.003	0.000	0.000
		df	330	330	330	330

^*∗∗*^Correlation is significant at the 0.01 level (2-tailed). ^*∗*^Correlation is significant at the 0.05 level (2-tailed).

**Table 2:** *R* ^2^ of BPNNs.

Day type	Working days			Nonworking days	
Station type	Cluster 1	Cluster 2	Cluster 3	Cluster 1	Cluster 2
*R* ^2^	0.92	0.92	0.96	0.90	0.94

**Table 3:** Abs error of random trials.

Day type	Working days			Nonworking days	
Station type	Cluster 1	Cluster 2	Cluster 3	Cluster 1	Cluster 2
Abs error = 0 (%)	81.2	80.3	74.9	74.6	70.6
Abs error ≤ 1 (%)	92.2	92.8	96.9	92.0	91.4

Abs error = 0 (%)	83.0	80.2	76.9	75.0	72.5
Abs error ≤ 1 (%)	93.5	93.5	98.3	92.6	93.4

Abs error = 0 (%)	73.7	71.9	70.4	72.1	70.3
Abs error ≤ 1 (%)	86.9	88.3	93.0	89.1	89.6

**Table 4:** *R* ^2^ of BPNNs under Scenario 1 and Scenario 2.

Different scenarios	Scenario 1		Scenario 2	
	Working days	Nonworking days	Cluster 1	Cluster 2
*R* ^2^	0.59	0.52	0.71	0.64

**Table 5:** Abs error of random trials under Scenario 1 and Scenario 2.

Different scenarios	Scenario 1		Scenario 2	
	Working days	Nonworking days	Cluster 1	Cluster 2
Abs error = 0 (%)	27.6	13.1	30.2	32.0
Abs error ≤ 1 (%)	60.8	48.5	64.9	64.4

Abs error = 0 (%)	27.2	11.9	30.3	33.4
Abs error ≤ 1 (%)	62.4	47.7	66.4	64.1

Abs error = 0 (%)	23.8	14.5	25.3	34.1
Abs error ≤ 1 (%)	58.9	47.4	62.1	63.4

## Data Availability

The data used to support the findings of this study were provided by the Wenzhou Urban Planning & Design Institute. Access to these data will be provided by the first author upon request, with permission of the Wenzhou Urban Planning & Design Institute.
